# From desolation to preservation: Investigating longitudinal trends in forest coverage and implications for future environmental strategies

**DOI:** 10.1016/j.heliyon.2024.e25689

**Published:** 2024-02-06

**Authors:** Muhammad Asif Khan, Sajid Ali, Muhammad Khalid Anser, Abdelmohsen A. Nassani, Khalid M. Al-Aiban, Shafiq ur Rahman, Khalid Zaman

**Affiliations:** a Department of Forestry & Wildlife Management, The University of Haripur, Haripur Khyber Pakhtunkhwa 22620, Pakistan; b School of Economics, Bahauddin Zakariya University, Multan, Pakistan; c School of Business, Xi'an International University, Xi'an 710077, Shaanxi, China; d Department of Management, College of Business Administration, King Saud University, P.O. Box 71115, Riyadh, 11587, Saudi Arabia; e Department of Public Administration, College of Business Administration, King Saud University, P.O. Box 71115, Riyadh 11587, Saudi Arabia; f Bahria University Law School, Bahria University, Islamabad, Pakistan; g Department of Economics, The University of Haripur, Haripur Khyber Pakhtunkhwa 22620, Pakistan

**Keywords:** Forest cover, Afforestation programs, Climate change, Environmental regulations, Population growth, Inbound FDI, Pakistan

## Abstract

Pakistan's forest cover is experiencing significant degradation in the ongoing efforts to combat climate change. The current state of the climate catastrophe is acknowledged. Nevertheless, there is a significant lack of readiness to tackle it effectively, especially regarding safeguarding the welfare of forthcoming generations. Pakistan bears significant relevance for future generations in this global crisis. The primary objective of this study is to examine the environmental difficulties faced by Pakistan and emphasize the critical need to safeguard its natural resources, considering the well-being of present and future generations. By using rigorous correlation and robust least squares regression methods, we investigate the complex interplay of financial aid, environmental legislation, precipitation, population growth, foreign direct investment, and afforestation within the time frame spanning from 1990 to 2022. The findings demonstrate that providing financial aid for afforestation initiatives significantly expands forested areas in Pakistan. Furthermore, the expansion of the population, the implementation of rigorous environmental restrictions, and the yearly amount of precipitation all play a role in the augmentation of forest coverage in Pakistan. Nevertheless, an alarming pattern of diminishing forest coverage over the years presents noteworthy obstacles. The importance of governance in promoting afforestation initiatives and sustainable development is highlighted by the emergence of adequate regulatory quality as a key factor. The average amount of precipitation has a discernible beneficial influence, underscoring the significance of climatic factors. The results above emphasize the need to implement cautious water resource management strategies and regulations responsive to climatic conditions. Based on these observations, the study proposes promoting sustainable agricultural and forest management, adopting a well-balanced strategy towards population expansion, implementing regulatory changes, and prudent use of water resources.

## 1 Introduction

Forests are essential to biodiversity conservation. Pakistan's forests are diverse, including mangroves and sub-alpine meadows. However, inefficient management has hindered forest coverage in recent decades. Increasing forest cover and reducing deforestation are urgent steps to preserve Pakistan's biodiversity. Pakistan's conservationists are working to increase forest coverage via the Billion Tree Afforestation Project and the Ten Billion Tree Tsunami Project. Many remote Pakistani villages rely on fuel wood for food. Therefore, deforestation remains a significant issue. Pakistan ranks 113rd in forest cover out of 143 nations [1]. Despite numerous challenges, now is the best time to promote forest conservation. The stated Programmes improve rural livelihoods and climate-smart landscape restoration and management [2]. The Bonn Challenge aims to restore 150 million hectares of deforested or damaged land by 2020. This effort now aims to rehabilitate 350 million hectares by 2030. The Billion Tree Afforestation Programme (BTAP) may help restore and improve the ecosystem in response to environmental degradation. The program's crucial monitoring and evaluation mechanism, administered by WWF Pakistan, fosters transparency and records development progress [3]. Harbi et al. [4] found that afforestation improves ecosystem quality by increasing forest and tree coverage. This benefits many living things and human society economically. Pakistan lost 170,684 ha of forest between 1990 and 2010. The reduction may be attributed to agricultural expansion or contraction, alpine pasture management changes, animal population, feeding methods, and increased wood extraction [5]. The data show that the BTAP improved social sustainability by 69% between 2014 and 2018. The program's 6.9 million USD net income in the three Khyber Pakhtunkhwa (KPK) districts shows its effect [6].

The growing worldwide population has caused deforestation, putting strain on forest ecosystems. Population growth threatens forest resources. The 7th Population and Housing Census-2023 by the Pakistan Bureau of Statistics found 241.49 million people in Pakistan, spanning a broad variety of geographic locations. The first computerized census showed a 2.55% population growth rate [7]. Rehman et al. [8] emphasized the global effect of population growth. To promote sustainable growth, proactive measures like afforestation and habitat preservation are needed. Forests provide regional livelihoods, economic benefits, and global ecological services. Human activities have put a burden on forest resources, leading to forest degradation, environmental degradation, and the loss of livelihood possibilities [9]. Due to unsustainable consumption and production, humanity faces climate change, biodiversity loss, and pollution [10]. Tropical forest resources, especially in underdeveloped nations, have declined due to poor management [11]. Nearly 880 million people use firewood or charcoal from trees for home energy. A large portion of this demography is female. After receiving international recognition, the Saudi Arabian government invited Pakistan to collaborate on an afforestation project with the goal of planting 10 billion trees in Saudi Arabia [12].

Due to its worldwide location, Pakistan is one of the most vulnerable nations to climate change. Plant life depends on sufficient moisture. The World Bank reported a reduction in precipitation from 392.53 mm in 2020 to 228.18 mm in 2021. Precipitation helps afforestation thrive. Pakistan is one of the first seven nations to experience climate change due to its location. Pakistan's contribution to global GHG emissions is less than 1%, yet the 2022 flood catastrophe affected 33 million people. Pakistan is one of the top ten nations hit by harsh weather. Afforestation, targeting 25–40% of a watershed area, can reduce flood damage by 20%. Effective water resource management and disaster risk reduction need ongoing monitoring of precipitation patterns [13]. Despite climate change's significant impact on precipitation patterns in northern Pakistan, more research is needed to understand spatiotemporal variations of precipitation within homogeneous climatic zones and their correlation with larger-scale mechanisms [14]. In vegetation-rich areas, evapotranspiration rises, raising atmospheric moisture. Increased moisture affects precipitation patterns. Many farmers have stopped farming owing to precipitation changes, resulting in financial losses. Pakistan is vulnerable to climate change due to its dry and semi-arid climate. Pakistan, like other countries, is subject to harsh weather patterns caused by climate change, which endangers food production and availability. The impact of annual precipitation on Pakistan's afforestation efforts is vital for making educated choices, allocating resources, and adjusting to changing climates. Forests absorb precipitation and improve soil penetration, reducing floods. Water flows are more sustainable, soil erosion is reduced, and soil quality is improved. Since 1990, severe precipitation occurrences in northern Pakistan have decreased in severity, frequency, and geographical extent [15]. This alteration affected local forests.

Foreign direct investment (FDI) helps Pakistan, which has limited financial resources, get vital financial resources. Rehman et al. [16] suggest that the Pakistan's government should improve infrastructure development by encouraging international investment. Pakistan, a developing nation, desires to increase forest density to combat climate change. The IPCC estimates that Pakistan's average temperature swings will be 1 °C higher than the global average by 2100. Pakistan has seen countless climate-related floods and disasters. The loss of arable land and necessary infrastructure and the inability to give enough incentives to individuals affected by these issues have hindered their ability to recover their land and rebuild their infrastructure. In this context, foreign organizations for funding Pakistan's climate adaptation efforts are appreciated. Pakistan received $77.8 million in finance on July 13, 2023, to improve Pakistan's climate change resilience by utilizing Ecosystem-based Adaptation and Green Infrastructure. These methods would merge flood risk management with water security. A US$66 million Green Climate Fund grant and $11.8 million co-financing are included. This financial assistance is predicted to help 687,336 direct recipients and 7,024,361 indirect beneficiaries. Relaxing environmental laws in emerging countries has had lasting effects on industry and development [17]. Losses and damages from the Pakistani floods earlier this year, caused by severe climate change, exceeded $30 billion. This event highlighted the significance of this vital problem again. According to Munir & Ameer [18], FDI may introduce unwanted technology to the receiving country, causing environmental harm. Thus, eco-friendly technology is crucial. A financing approach to plant 10 billion trees may entail exploring foreign money options. Environmental initiatives may be funded by the Green Climate Fund, the 10.13039/100011150Global Environment Facility, the International Finance Corporation, and environmental financial instruments like the Asian Development Bank's and China's Green Bonds. Given the programs' broad breadth and ability to benefit humanity and the environment, the method above may be critical [19].

From the provided discussion, the following research questions have arisen:

1. ***Does financial assistance and environmental regulations enhance environmental quality by promoting increased afforestation programs within a given country?*** This research question is crucial as it investigates whether financial support and environmental regulations can create a powerful synergy for promoting afforestation programs. Understanding how economic incentives and regulatory frameworks work together to enhance environmental quality can guide policymakers in designing effective strategies for sustainable development.
2. ***How does annual precipitation influence the success and growth of afforestation efforts in Pakistan?*** The significance of this research lies in its exploration of the role of annual precipitation in shaping the outcomes of afforestation initiatives in Pakistan. Identifying the relationship between water availability and afforestation success is vital for making informed decisions about resource allocation and adapting afforestation efforts to changing climate patterns.
3. ***To what extent do population growth rates and FDI inflows impact the progress and outcomes of afforestation initiatives in Pakistan?*** This research question is crucial because it addresses the complex interplay between population growth, foreign investment, and afforestation outcomes. By investigating how these factors influence each other, the research contributes to a better understanding of how economic and demographic trends impact environmental sustainability and the success of afforestation projects in Pakistan.

The study has the following research objectives:

1. Assess the effectiveness of combining financial assistance with environmental regulations in promoting afforestation programs in a country.
2. Identify strategies for adapting afforestation projects to varying annual precipitation levels, considering potential climate change effects.
3. Examine the impact of population growth rate and FDI inflows on the advancement and achievements of afforestation projects in Pakistan.

The variables estimations were acquired using the robust least squares regression in this examination. This analytical tool addresses model outliers to provide accurate parameter computations. The given strategy improves the statistical methodology by noticing the study's critical variables.

The study is structured into the following sections: the literature review is presented in section 2 after the introduction. The theoretical framework is discussed in section 3. Data and methodology are presented in section 4. Results are discussed in section 5. The final section concludes the study.

## 2 Literature review

It is imperative that we remain steadfast in our commitment to safeguard and preserve our natural resources. In light of the prevailing data concerning Pakistan's forest coverage, a comprehensive approach becomes indispensable in addressing the challenges that obstruct the progress of afforestation endeavors within the country. The objective of this study is to establish a conceptual framework aimed at augmenting forest cover, conserving biodiversity, and procuring funding at both the national and international levels to facilitate the expansion of afforestation and reforestation initiatives in Pakistan. This multifaceted approach seeks to create a landscape capable of withstanding climate change mitigation measures, while simultaneously meeting the growing public demand for sustainable development and fostering collaboration among stakeholders. This examination underscores the deficiencies in current government policies in mitigating these challenges and underscores the urgency of formulating sustainable development strategies. Furthermore, the collection of biodiversity data is emphasized as pivotal for sustainable development and for informed decision-making pertaining to the preservation of natural resources, as articulated by Ali et al. [20] study. Given Pakistan's precarious status in terms of forest coverage, the focus should shift from the formulation of new afforestation policies to the meticulous implementation of existing policies. This study explores dynamic and innovative variables, hitherto underexplored in prior research on afforestation in Pakistan, notwithstanding the predominant reliance on contemporary data in most recent studies. The literature review is categorized into three distinct sub-themes, each outlined as follows:

### 2.1 Financial assistance for afforestation programs

The provision of financial support is imperative to incentivize afforestation initiatives in underdeveloped nations. A comprehensive assessment by Zada et al. [2] reveals the commendable progress of Pakistan's Billion Tree Afforestation Program, attaining its objectives of enhancing the well-being of rural populations and proficiently managing the landscape with a climate-conscious approach. The adoption of agroforestry practices for sustainable development and the cultivation of farmer collaboration in afforestation initiatives are found to be influenced by a multitude of factors. Ullah et al. [21] investigation scrutinizes the adoption of agroforestry in Pakistan, with a particular focus on the influence of green growth initiatives such as the Billion Trees Afforestation Project. This research reveals that early adopters are primarily motivated by the protection of crops from environmental hazards, while their late counterparts seek diversification of income sources. The adoption rates are positively affected by variables like age, education, and community involvement, whereas political conflicts and land tenure insecurity exert negative effects. The study recommends the implementation of collaborative strategies and the engagement of educated, senior farmers to promote agroforestry practices in the region. While several countries have witnessed a rapid expansion in forest coverage, Chakma et al. [22] study on the Chittagong Hill Tracts in Bangladesh, employing Landsat images from 1998, 2008, and 2018, unveils a noteworthy 58.03% increase in forest cover from 1998 to 2018. The study attributes this growth to community forestry practices and the formulation of national forest strategies. Nonetheless, it emphasizes the imperative of revising forest policies to strike a balance between meeting the population's lumber and timber needs and environmental conservation, especially in the context of climate change.

The study by Ullah et al. [23] delves into the Billion Trees Afforestation Project (BTAP) within Pakistan's Khyber Pakhtunkhwa region, specifically examining the Dir Kohistan Forest division. Employing a mixed-methods approach, the research underscores the positive impact of establishing community-based organizations, fostering increased participation, and consequently, enhancing livelihoods. However, this study also reveals a need for further exploration and resolution of the challenges hindering successful community-based participation in landscape co-management, particularly in the context of the BTAP in KPK, Pakistan. Tahir et al. [24] accentuate the rising interest in integrated landscape approaches to mitigate deforestation and forest degradation (REDD+) within the UNFCCC. Their study employs a multi-criteria decision-making model to assess the effectiveness of REDD + implementation and forest sustainability across South Asian countries. Nepal exhibits the highest sustainability scores, while Pakistan reports the lowest, affording valuable insights for policy-based indicators aimed at promoting sustainable forest management and carbon emissions reduction in developing countries. Observing the substantial contributions of our country's afforestation program to sustainable development, Rauf et al. [25] scrutinized the impact of the BTAP on local households in KPK Province, Pakistan. Collecting data from 360 residents and employing statistical methods, including ordered logit and ordinary least squares, the study revealed a significant positive influence of BTAP on rural livelihoods. Households linked to BTAP experienced a 4% increase in income and a 35% surge in assets. Nevertheless, the research gap in this study is the absence of an in-depth exploration of the specific mechanisms and factors driving BTAP's impact on household income and asset growth within the region, thereby necessitating further research in this domain. Conversely, Khan et al. [6] investigated the socioeconomic impact and local perception of the BTAP in KPK Province, Pakistan. Their research illuminates the commendable and favorable overall social impact of BTAP, contributing to a 69% increase in social sustainability from 2014 to 2018. Furthermore, it positively influences the economic conditions of rural households, culminating in a cumulative net income of 6.9 million USD across the three districts of KPK. This study emphasizes the continuous endeavors required to augment rural household income and environmental protection. Nazir et al. [26] concentrate on the BTTAP in Pakistan's KPK province. Employing a system dynamics model constructed from project data, the study predicts a projected 3.29% increase in forest area, surpassing the initial 2% target. However, certain categories of afforestation lag behind, warranting special attention. In light of the comprehensive discussion, the first hypothesis of this study is articulated as follows:

H1 Increased financial support for afforestation initiatives will lead to a significant rise in afforestation and reforestation rates, consequently yielding improved outcomes in ecological restoration within the designated areas.

### 2.2 Environmental Regulations Stringency for afforestation programs

Since 1955, Pakistan has implemented various policies with distinct objectives to enhance forest cover; however, deforestation has persistently limited these efforts. Given the evolving dynamics attributed to climate change, there is an urgent need to revise the KP Forest Ordinance of 2002. According to Doelman et al. [27], afforestation emerges as a cost-effective strategy for mitigating climate change, holding the potential to reduce 4.9 GtCO2/year by 2050, at a cost of $200/tCO2. Nonetheless, it is critical to acknowledge associated risks, including potential reduced ambition in other sectors, challenges in regions with weak governance, and the substantial land requirements (up to 1100 Mha), which may lead to increased food prices and food security concerns. These risks necessitate careful management and trade-offs for sustainable mitigation, particularly when targeting climate objectives of 2 °C or 1.5 °C. Mehmood et al. [28] investigated the influence of green industrial transformation on reducing carbon intensity in Pakistan over the period from 1975 to 2020. The findings demonstrate that green industrial transformation is associated with reduced carbon emissions, albeit with factors such as FDI, technological innovation, and R&D investment contributing to increased carbon emissions. These outcomes support the pollution haven hypothesis. Consequently, it is suggested that Pakistan should prioritize a green industrial revolution to attain environmental sustainability and fulfill its environmental objectives. Ali [29] highlight Pakistan's significant role in China's Belt and Road Initiative (BRI), with a specific focus on the CPEC, representing a substantial $46 billion investment aimed at developing energy infrastructure, communication networks, and industrial zones. The research emphasizes the Pakistani government's optimistic outlook regarding CPEC energy projects, considering them vital for addressing the country's energy crisis. Similarly, Khalid et al. [30] stress the imperative of sustainable development in response to the adverse environmental impacts resulting from human-induced climate change. Their research scrutinizes the environmental consequences of infrastructural developments within the CPEC. Employing a qualitative approach and official documents, the study identifies three major concerns: CO2 emissions from coal-fired power plants, deforestation for road infrastructure, and increased vehicle emissions on the Karakorum Highway. This research underscores the necessity of reevaluating the environmental costs associated with CPEC and advocates for enhanced economic and legal cooperation between Pakistan and China to address climate change issues through the integration of environmental laws into CPEC projects. The overarching theme in these discussions is the recognition that climate change is an inescapable reality, compelling nations to adapt and collaborate in the pursuit of shared goals, particularly in the context of environmental laws and regulations.

Hasan et al. [31] investigate climate change mitigation efforts within rapidly growing developing countries, focusing on Bangladesh's policies following the 2016 Paris Agreement. Despite government initiatives aimed at reducing GHG emissions, recent trends suggest an increase in emissions, raising concerns about the effectiveness of these policies. The study assesses these policies using various indicators, ultimately revealing limited impacts on energy decarbonization, energy demand management, and the improvement of emissions sinks. Some policies align with national development goals, while others, such as coal-based electricity generation and forest biomass utilization, exhibit little synergy. Lei et al. [32] evaluate the role of environmental and energy-related technological innovations in the pursuit of global environmental sustainability objectives across South and Southeast Asia. The study underscores the direct and indirect role of good governance in promoting sustainability, whereas urbanization and economic globalization are identified as potential impediments to progress. On a broader scale, Southeast Asia exhibits more promise in attaining environmental sustainability, leading to valuable policy recommendations derived from these findings. Similarly, Anwar et al. [33] emphasize Pakistan's rich biodiversity, spanning multiple zoogeographic regions and facing increasing threats from climate change. The study underscores Pakistan's vulnerability to climate change, existing policy frameworks, and proposes various measures, including community awareness programs, buffer zone management, afforestation, and research, to enhance biodiversity conservation and climate adaptation. Although Pakistan possesses substantial potential for climate resilience, the negative consequences of climate disturbance have an adverse influence on the country's economy and overall sustainability. Abbas et al. [34] conducted a study highlighting the global challenges arising from limited investments in land degradation neutrality (LDN), emphasizing the adverse environmental and socio-economic impacts of this insufficiency. The study endeavors to achieve Sustainable Development Goal 15.3 by 2030, focusing on Pakistan's 14 degraded districts. The research identifies government commitment, coordination mechanisms, and public-private partnerships as crucial enablers of LDN. Challenges include raising awareness about LDN and assessing market risks. The benefits of such endeavors include fostering a green economy and ecological sustainability. Ashraf et al. [35] conducted a study on the TBTTP in Pakistan, a high-profile tree-planting initiative with a substantial $700 million budget. Despite claims of planting over a billion trees and creating 165,000 jobs, the study reveals a pattern of unequal distribution of benefits, predominantly favoring the affluent while excluding herders and landless individuals. Furthermore, Durani et al. [36] investigate the Pollution Haven Hypothesis (PHH) in BRICS nations, exploring the relationship between FDI, air pollution, and environmental regulations. The study employs BRICS' COP26 goals and the SDGs agenda for 2030 as a backdrop, utilizing panel data from 2000 to 2020, employing the PMG/PARDL model. Their findings affirm the existence of the PHH, signifying that lower regulatory stringency may attract pollution-intensive FDIs. This underscores the need for flexible environmental policies that can stimulate FDIand support SDG achievement, while also highlighting the potential for stricter regulations to encourage more environmentally responsible FDIs. Wang et al. [37] delve into the impact of stringent environmental policies and the transition to renewable energy on environmental quality within BRICS economies from 1990 to 2019. The study demonstrates that stringent environmental policies effectively reduce carbon emissions in these economies, with renewable energy adoption inversely correlated with CO2 emissions. Economic growth and industrial value-added, conversely, contribute positively to carbon emissions. Notably, the combined implementation of stringent policies and the transition to renewable energy exhibits a more substantial impact on carbon reduction. In light of the comprehensive discussion, the second hypothesis of this study is presented as follows:

H2 The effectiveness and sustainability of afforestation initiatives, as determined by the growth and health of planted trees and their long-term effects on regional ecosystems, positively correlate with the tightening of environmental regulations.

### 2.3 Climate change, population growth, and FDI inflows for afforestation programs

The prioritization of community needs over fundamental rights often places significant stress on forest resources due to population growth, leading to the unfortunate consequence of illegal deforestation. A study by Hussain et al. [13] examined temporal deforestation patterns in the remote mountainous region of Tribal District Kurram, Pakistan, spanning the years 1972–2019. Their investigation unveiled a substantial 48% reduction in forest cover within this period, with population growth and improved accessibility emerging as key contributing factors to this deforestation. This underscores the intricate interplay between socio-economic factors and the biophysical environment. Dat & Le [38] delve into the challenges faced by FDI enterprises in Vietnam, specifically amidst the evolving COVID-19 pandemic. By employing quantitative methods and surveying 700 foreign investors, the research identifies three pivotal factors (investment policy, investment environment, and the quality of human resources) influencing FDI attraction, particularly within Vietnam's green economy. The essence of this research lies in guiding policymakers to enhance capital attraction efficiency and foster green economic growth, all within the overarching context of climate change and international integration. In line with this global perspective, Khan et al. [39] draw attention to Pakistan's vulnerability to climate change, despite its minimal contribution to global greenhouse gas emissions. Climate change poses a formidable challenge to the economic, social, and environmental dimensions of sustainable development, affecting key sectors such as food, water, energy, biodiversity, and exacerbating climate-related hazards like floods and droughts. While the research raises awareness about the serious dangers and vulnerabilities posed by climate change in Pakistan, it remains a subject of concern that it does not encompass a comprehensive examination of the strategies deployed to manage and mitigate these risks. This deficiency represents a research gap that warrants further exploration. In the study conducted by Ali et al. [40], the focus shifts from rural livelihoods to investigating the macroeconomic impact of forest plantations on Asia's GDP per capita. Leveraging data spanning from 1990 to 2019, the research demonstrates that forest plantations, coupled with variables such as total exports, agricultural growth, and FDI, exert a positive and significant influence on GDP per capita across Asian countries. The research further suggests that the promotion of forest plantations can not only enhance GDP per capita but also contribute to long-term environmental benefits for the region's economies.

Khan et al. [41] undertake a comparative assessment of land use and land cover changes in Islamabad (a planned city) and Rawalpindi (an unplanned city), spanning the period from 1990 to 2021. The study reveals a substantial expansion of built-up areas and a concurrent decrease in bare land in both cities, with these changes strongly correlating with population growth. The implications of these findings are critical for the formulation of land use and urban sprawl management policies that align with the broader objectives of sustainable development. Zaheer et al. [42] conducted a comprehensive study on the TBTTP in Pakistan. This initiative aims to combat climate change and address water scarcity by planting ten billion trees in regions prone to deforestation and affected by drought over a five-year period. The program envisions enhancing forest cover, ensuring increased water availability, mitigating soil erosion, bolstering environmental resilience, creating employment opportunities, and promoting eco-tourism. A favorable environment conducive to forest growth can have a notable positive impact on Pakistan's economy. Najeeb et al. [43] conduct an analytical study highlighting the indispensable role of forests within Pakistan's economy and environment. This study emphasizes the pressing need for increased forest conservation while exploring the historical and contemporary importance of forests in the country's economic development and their influence on atmospheric conditions. In a political context, Syed et al. [44] investigate one of Pakistan's political parties and its Clean and Green initiative in response to the global challenge of climate change. Employing a qualitative research methodology, they find that the policies of this political party yield positive impacts at both the national and international levels. This underscores the critical role of regional and local governments as essential agents for implementing effective environmental strategies. The study's final hypothesis is as follows:

H3 There will be a positive correlation between FDI inflows and the implementation of afforestation programs in affected regions as global population growth and climate change intensify, showing that international capital is increasingly going toward addressing environmental issues and sustainable development.

The study makes a significant contribution by evaluating the influence of financial assistance for afforestation programs on forest coverage in Pakistan. While previous investigations focused primarily on the primary dataset from various cities in the Khyber Pakhtunkhwa Province (KPK) [6,45], this research pioneers an inclusive analysis by incorporating a secondary dataset covering the entirety of Pakistan. This broader approach aims to yield more comprehensive insights into the nationwide impact. Additionally, the analysis incorporated environmental regulations Stringency, a vital factor influencing forest coverage and afforestation programs. Unlike previous studies that primarily addressed specific action programs rather than institutional measures supporting conservation [33,46], this research provides a more holistic perspective. Furthermore, the study uniquely assesses the simultaneous impact of climate vulnerability and foreign investment, factors previously examined separately in the context of forest conservation programs [47,48]. The integration of these variables enables a thorough examination of the role of financial development in the context of climate financing and its impact on Pakistan's forest coverage.

## 3 Data source & methodology

### 3.1 List of variables and their measurement

Below is a list of variables used in our study, along with their respective measurements.

- Dependent Variable:
1. **Forest Area:** This variable represents the total land area covered by forests in Pakistan over time, serving as a direct measure of changes in forest cover resulting from afforestation programs. Forest area is measured as a percentage of the total land area and is sourced from the World Development Indicators [49].
- Independent Variables
1. **Financial Assistance for Afforestation Programs:** This variable represents Pakistan's economic performance, as measured by GDP, which can influence the financial resources available for afforestation programs. It is approximated using “Agriculture, forestry, and fishing, value added (% of GDP)" as a proxy and is expressed as a percentage of GDP, obtained from the WDI [49].
2. **Population Growth Rate:** This variable accounts for the impact of population growth on the demand for resources, including forest products, potentially influencing afforestation strategies and outcomes. Population growth rate is measured as an annual percentage and is sourced from the WDI [49].
3. **Environmental Regulations Stringency:** This variable represents an index indicating the strength of environmental regulations and policies in Pakistan over time, potentially affecting the success of afforestation programs. The study employs “Government Regulatory Quality: Estimate” as a proxy variable for environmental regulation stringency, indexed on a scale ranging from −2.5 to 2.5. Data for this variable is derived from the WDI [49].
4. **Annual Precipitation:** This variable signifies the amount of rainfall in Pakistan, which is crucial because afforestation programs are influenced by water availability, impacting tree growth and survival. It is measured in terms of average precipitation in depth (mm per year) and is obtained from the Climate Change Knowledge Portal [50].
5. **Foreign Direct Investment (FDI):** Foreign investments in afforestation initiatives can influence the resources available and the implementation strategies. The unit used for FDI is (% of GDP). Data for this variable is sourced from the WDI [49].

### 3.2 Data source

The data for our study is drawn from two primary sources: the World Development Indicators [49] and the Climate Change Knowledge Portal [50] databases. These datasets provided information on the variables essential for our study, which focuses specifically on Pakistan. Our dataset comprises one dependent variable and five independent variables, covering the years from 1990 to 2022.

### 3.3 Justification for the study in Pakistan

Pakistan faces significant challenges related to climate change, and it is essential to evaluate these issues within the country's specific context to formulate sustainable policies. The study emphasizes the long-term impact of weak political institutions on economic development [51]. A comprehensive understanding of Pakistan's economy is crucial to develop robust policies that promote forest area, enhance livelihoods, and create jobs. Pakistan stands at a critical juncture in a changing global landscape, and decisions made today will shape the nation's future. Forests are crucial for mitigating global temperature rise. Forest degradation due to urbanization and industrial growth has contributed to the increase in global average surface temperature [41]. Researching and understanding Pakistan's unique dynamics is necessary for a more promising and sustainable future. Adoption of green design, procurement, and construction practices can enhance both environmental and economic performance [52]. In-depth analysis of GDP trends can optimize resource allocation, boost productivity, and encourage innovation. Pakistan's vulnerability to climate change impacts necessitates action. Increasing forest area, as opposed to rapid deforestation, is essential [53]. Attracting foreign investment is vital for economic development, while analyzing annual precipitation trends can help manage forest resources sustainably. Policies and programs are being developed to increase forest area, but significant progress remains elusive. Ensuring a prosperous, sustainable, and equitable future for all Pakistanis is imperative.

### 3.4 Theoretical framework

#### 3.4.1 The tragedy of the commons

The tragedy of the commons is a concept from economics and environmental science that is applicable to forestry and resource management. When forests are managed as a communal resource, they may be overused and degraded. Notably, the nationalization of forests during the 20th century occasionally disrupted established management practices, leading to a “tragedy of the commons” [54]. According to Garrett Hardin's Tragedy of the Commons theory, resources used collectively tend to become overused or degraded. In forestry, sustainable forest resources are a prominent common pool resource of global significance. Historical actions have resulted in the misuse of Pakistan's forest resources, jeopardizing the rights of neighboring communities. Sustainable development through afforestation can help address this challenge. Local populations often exploit forest resources for various reasons. In some instances, involving local communities in forest management decisions can lead to more sustainable outcomes. Joint forest management is essential for limiting the burden on forests for sustainable development in Pakistan. The loss of habitat leads to a decrease in biodiversity. Deforestation is a significant contributor to climate change. Unlawful deforestation has resulted in catastrophic disasters in Pakistan's history. Individuals often prioritize short-term economic gains over long-term sustainability, contributing to forest degradation. Pakistan is a country with rich biodiversity, but this is threatened by the ongoing environmental changes. Pakistan is highly vulnerable to climate change, ranking as the 6th most affected country in the German Watch Report 2020 and the 8th most vulnerable nation to climate crises according to the Global Climate Risk Index. Regulations and monitoring programs are essential for ensuring sustainable and environmentally sound forest management. The government should provide alternative energy sources to reduce reliance on trees and address the root causes of deforestation [40]. Clarifying ownership and property rights over forested areas can promote responsible management and long-term sustainability. The Green Economy is a sustainable development paradigm supporting ecologically and socially sustainable growth [55]. Studying soil carbon changes due to afforestation and reforestation, as well as the effects of deforestation on soil carbon levels, is crucial [56]. Increasing awareness of the value of forests and the negative effects of overexploitation can promote responsible forest management. Forests contribute to long-term sustainability [57].

#### 3.4.2 Institutional analysis & development framework

The Institutional Analysis and Development (IAD) framework, developed by Elinor Ostrom and others, is a valuable tool for understanding the governance and management of common-pool resources like forests. It allows for the examination of institutions and processes that affect forest management, governance, and utilization. Notably, there are distinct policies in place within the Forest Department and Pakistan's Ministry of Climate Change. However, one of Pakistan's primary challenges in implementing climate change policies is the lack of political commitment and policy prioritization [58]. The Pakistani Ministry of Climate Change oversees the forest department, which used to be under federal government jurisdiction. Later, forestry became a provincial matter, allowing each province to manage its own forests. Various organizations and NGOs, including the 10.13039/100016195UNDP, UNEP, IUCN, and 10.13039/501100010732WWF Pakistan, contribute to the forestry sector through funding, monitoring, training, and afforestation initiatives. Each level of the department has specific territorial responsibilities. Collaborative efforts between the forest department, local communities, and NGOs are essential for the effective protection and expansion of forest cover. Urban planning, particularly safeguarding urban forests, is vital for mitigating flood impacts in densely populated cities. Pakistan has lost a significant portion of its forest cover in the past two decades. The impacts of climate change often receive inadequate attention and funding due to political instability and frequent changes in administration. The role of institutions in influencing access, utilization, and management of forest resources is crucial [59]. Good governance is crucial for Pakistan's sustainability. Protected areas have various objectives that sometimes compete, including tourism, community livelihoods, and biodiversity preservation. However, it is challenging to determine which governance strategies are most effective in fulfilling these objectives [60]. Innovative governance options are needed to adapt to changing conditions and pressures on society and ecosystems [61]. Planning is essential for protecting natural resources and promoting sustainable usage. Pakistan is transitioning towards renewable energy to address its electricity shortage [62]. Understanding the intricate relationship between power and formal and informal institutions is critical for improving local and regional government participation and service delivery. Fostering inclusive governance and empowering local communities are essential for effective service delivery and participation.

### 3.5 Econometric framework

To comprehensively explore the complex relationship between afforestation efforts and sustainable development in Pakistan, our study employs a robust econometric methodology. This approach combines economic and environmental data to identify the primary variables influencing changes in forest cover, shedding light on the broader implications for the socio-economic development and environmental well-being of the country. The fundamental theoretical underpinnings of the primary linear modeling approach, known as OLS, are based on the idea that the relationship between dependent and independent variables can be described by a simple linear equation, where the values of the dependent variable are estimated based on the values of independent variables [63]. Ordinary Least Squares (OLS) is a standard method for estimating the coefficients of linear regression equations that describe the relationship between one or more independent quantitative variables and a dependent variable. The OLS method is essential for finding the best-fitting linear coefficients within regression equations and is a key component of our study [64]. OLS estimators are sensitive to the presence of observations that deviate significantly from the regression model. Ordinary least squares estimators are unbiased and have the lowest variance, making them the main focus of this study among all linear unbiased estimators [65].

### 3.6 Outlier detection methods

Four methods are used to detect outliers in the data: RStudent, DFFITS, COVRATIO, and Hat Matrix.

#### 3.6.1 RStudent

Identifying outliers using RStudent's method is a critical analytical tool. This approach enhances sustainable afforestation methods and policy recommendations for Pakistan's environmental conservation efforts by pinpointing and analyzing data outliers related to changes in forest cover.

#### 3.6.2 DFFITS

The study aims to identify the outliers by DFFITS method to improve the precision and reliability of its conclusions, ultimately leading to more efficient strategies for sustainable afforestation and forest management in Pakistan.

#### 3.6.3 COVRATIO

In order to identify anomalies, the COVRATIO approach is used. In order to find important data points that can affect Pakistan's afforestation activities in relation to sustainable development objectives, this technique is vital.

#### 3.6.4 Hat Matrix

One of the most important tools for finding outliers is the Hat Matrix. The undertaking aims to use this matrix to identify and analyze key data points that might significantly change the way forest cover is measured in Pakistan. The final goal is to provide an accurate and thorough evaluation of afforestation programmes for sustainable development.

### 3.7 Leverage plots in robust least squares regression

The leverage plots provide insights into the influence of specific data points on the regression results, aiding in the detection of potential outliers or significant observations that could impact the model's accuracy. Researchers can enhance the reliability and robustness of their regression analysis, contributing to a more thorough evaluation of the variables affecting changes in forest cover in Pakistan.

### 3.8 Robust least squares (RLS) regression

Robust least squares encompass various regression techniques that are resilient and less sensitive to outliers. These techniques include M-estimation [66], S-estimation [67], and MM-estimation [68]. Each approach has its own emphasis:

• **M-estimation:** This approach addresses situations where the dependent variable significantly deviates from the regression model (large residuals), accounting for these outliers.
• **S-estimation:** S-estimation is a computationally demanding method that focuses on high-leverage outliers in the independent variables.
• **MM-estimation:** MM-estimation combines both S-estimation and M-estimation. S-estimation is initially performed, and then M-estimation is conducted using the results of S-estimation as a starting point. MM-estimation addresses outliers in both the dependent and independent variables.

Table 1 presents a comparison of various statistical techniques concerning different criteria used for employing the RLS estimator.

**Table 1:** Econometric technique comparison across various criteria.

Criteria	RLS	OLS	2SLS	GMM	ARDL
Resilience to Outliers	Highly robust, minimal impact of outliers on estimates	Susceptible to distortion in the presence of outliers	May be affected by outliers, impacting reliability	Affected by outliers, may lead to biased estimates	Relatively robust, but sensitivity to outliers can occur
Parameter Estimation	Efficient parameter estimation even with influential data points	Prone to biased estimates when outliers are present	Efficient, but sensitive to model misspecification	Efficient, but sensitive to model assumptions	Efficient parameter estimation, but sensitive to lag structure
Model Stability	Stable performance in the face of data fluctuations and extreme values	May exhibit instability or sensitivity to outliers	Sensitive to model specification, potential instability	Susceptible to instability with model over-specification	Stable, but can face issues with over-differencing
Distribution Assumptions	Non-parametric, fewer assumptions about data distribution	Assumes normality, may be restrictive	Requires normality assumptions, may limit applicability	Limited distribution assumptions, but subject to model misspecification	Relies on normality, potentially limiting flexibility
Applicability to Non-Normal Data	Suitable for non-normal data distributions	Assumption of normality can limit applicability	Limited flexibility with non-normal data	Moderately flexible with non-normal data	Moderately flexible with non-normal data
Computational Complexity	Low computational complexity, suitable for large datasets	Moderate computational complexity	Moderate computational complexity	Moderate to high computational complexity	Moderate computational complexity
Handling Heteroskedasticity	Robust to heteroskedasticity, providing reliable results	May struggle with heteroskedasticity data	Requires additional techniques for heteroskedasticity	Sensitive to heteroskedasticity, potential bias	Sensitive to heteroskedasticity, may require adjustments

Equation (1) shows the RLS based equation to estimate the model for robust policy inferences, i.e.,

(1) $$FC=\varphi_{0}+\vartheta_{1}AFF+\vartheta_{2}PG+\vartheta_{3}GRQE+\vartheta_{4}AP+\vartheta_{5}FDI+\zeta$$

where, FC shows Forest area, AFF shows Agriculture, forestry, and fishing, value added, PG shows Population growth, GRQE shows Government Regulatory Quality Estimates, AP shows Average precipitation in depth, FDI shows FDI net inflows, and ƹ shows error term.

## 4 Results & discussion

Commencing with an elucidation of descriptive statistics applied to the variables, which facilitates the observation of trends specific to each variable, this section includes an explanation of various statistical approaches. The size and direction of relationships between the mentioned variables are then determined through correlation analysis. The subsequent step involves conducting an ‘influence statistics' test, with the primary objective of identifying potential outliers in the provided model. An examination of ‘leverage plots,’ strategically employed to detect probable outliers within both the dependent and explanatory variables, ensues. Upon identification of potential outliers in the variables, a thorough examination utilizing the robust least squares test is undertaken. Renowned for its effectiveness in addressing such abnormalities, this test aids in generating impartial, consistent, and reliable parameter estimations. These specific analytical techniques are employed to enhance the accuracy of our forecasts regarding the variables influencing the change in forest cover in Pakistan, leading to a more comprehensive understanding of regional efforts to promote sustainable development. Through the application of these statistics, a deeper comprehension of the variables impacting changes in forest cover in Pakistan is attainable. For each variable, skewness and kurtosis values offer additional insights into the distribution and shape of the data. These numerical findings furnish essential quantitative data for the research, facilitating a thorough exploration of the factors influencing the transformation of forest cover in Pakistan within the context of sustainable development endeavors.

Table 2 summarizes key statistics pertaining to sustainable development and forest cover change in Pakistan as part of our research study. The data reveals that the average forest cover in Pakistan stands at approximately 5.372%, displaying moderate variability within a range from 0.187% to 6.407%. Afforestation efforts contribute to an average increase in forest cover by about 23.123%.

**Table 2:** Descriptive statistics.

Methods	FC	AFF	PG	GRQE	AP	FDI
Mean	5.372	23.123	2.265	−0.644	294.223	1.030
Maximum	6.407	25.617	3.297	−0.479	392.530	3.668
Minimum	0.187	20.677	1.204	−1.049	187.520	0.355
Std. Dev.	1.049	1.152	0.622	0.126	60.849	0.810
Skewness	−3.702	0.367	−0.0003	−1.053	−0.005	2.157
Kurtosis	19.311	2.839	1.841	4.484	1.747	6.781

Population growth rates average 2.265%, showing a range from 1.204% to 3.297%, indicating some stress on afforestation programs in influencing forest cover change. The estimate of Government Regulatory Quality (GRQE) indicates an average reduction of −0.644, with low variability. Similarly, the average annual precipitation is 294.223 mm, ranging from 187.520 mm to 392.530 mm. Annual precipitation plays a role in affecting the amount of forest cover. FDI averages 1.030%, ranging from 0.355% to 3.668%, showcasing diversity in the changes to forest cover. These data provide vital insights into the dynamics of sustainable development and changes in forest cover in Pakistan, facilitating thorough research and the development of policies. The statistical examination of the variables affecting the change in forest cover in Pakistan underscores the necessity of targeted policies. While the considerable variability in population growth and FDI emphasizes the significance of adaptive policies to address demographic and economic concerns, the adversely skewed distribution of forest area argues for targeted afforestation operations in regions with lower cover. To enhance the sustainability of afforestation activities and consider the impact of precipitation depth on water resource management, policymakers should prioritize regulatory adjustments. Table 3 shows the correlation matrix estimates.

**Table 3:** Correlation matrix.

Variables	FC	AFF	PG	GRQE	AP	FDI
FC	1					
	–					
AFF	0.178	1				
	0.319	–				
PG	0.122	0.431	1			
	0.496	0.012	–			
GRQE	−0.015	−0.146	0.252	1		
	0.930	0.415	0.156	–		
AP	−0.134	−0.178	−0.053	0.362	1	
	0.456	0.321	0.767	0.038	–	
FDI	0.110	−0.304	0.009	0.315	0.240	1
	0.538	0.084	0.958	0.073	0.176	–

**Fig. 1:**
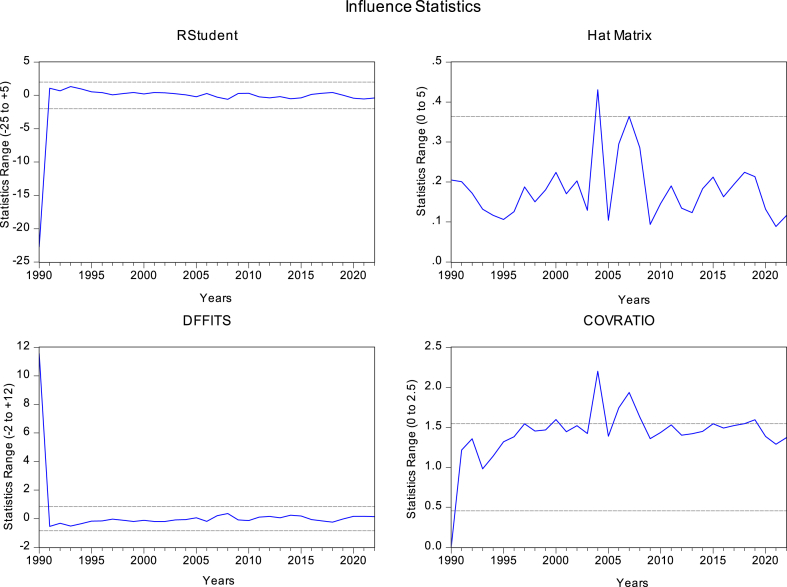
Influence statistics.

Source: Author's estimates. Black lines represent confidence intervals around the regression line. Blue line represents the estimated regression line.

The variable FC exhibits a positive association with AFF, PG, and FDI, indicating that larger contributions to GDP from agriculture, forestry, and FDI are correlated with larger forested areas. Conversely, it shows negative correlations with AP and GRQE, suggesting that declining forest cover is linked to more FDI, weaker regulatory quality, reduced precipitation, and faster population growth. Furthermore, it indicates that higher population growth and better regulatory quality coincide with a stronger contribution of these sectors to the economy. FDI positively correlates with FC, signifying that higher FDI is associated with increased forest area. AFF, measuring the contribution of agriculture, forestry, and fishing to Pakistan's GDP, positively correlates with FC and exhibits negative correlations with GRQE, AP, and FDI. PG displays negative correlations with AP and a positive correlation with GRQE and FDI. GRQE demonstrates negative correlations with FC and FDI, suggesting that higher regulatory quality is associated with a larger forest area and a stronger contribution of agriculture, forestry, and fishing to the GDP. These relationships shed important light on the complex variables influencing Pakistan's changing forest cover, forming the basis for the analytical framework of the study and enabling a greater understanding of the dynamics of sustainable development in Pakistan through afforestation. Fig. 1 provides valuable insights into potential outliers within the dataset.

**Fig. 2:**
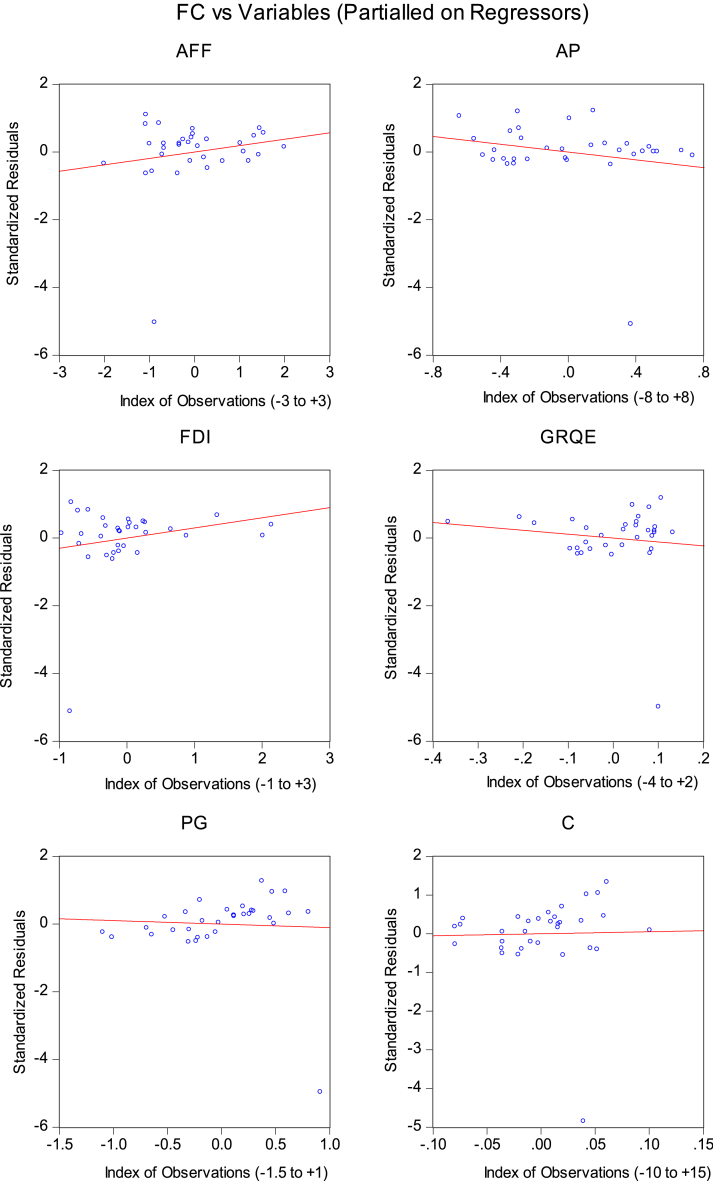
Leverage plots.

Source: Author's estimates. Red line represents the "Cook's distance” threshold. The blue circle values represent the individual observations.

The analysis uncovers several outliers detected through various statistical methods. Specifically, one outlier is identified by RStudent, indicating a data point that significantly deviates from the expected pattern. Another outlier is detected by DFFITS, suggesting a point with a disproportionate influence on the statistical model. The Hat Matrix identifies two outliers, highlighting cases with excessive leverage in the regression analysis. Furthermore, six outliers are flagged by COVRATIO, implying instances where the variance-covariance structure of the data is significantly affected by these data points. Identifying and understanding these outliers can refine the analysis and improve the accuracy of policy recommendations. Investigating the characteristics and possible causes of these outliers is advisable because they could provide insight into exceptional circumstances or factors influencing Pakistan's FDI, agricultural practices, population dynamics, and precipitation patterns. This data holds significant importance in guiding decision-making and shaping successful strategies for achieving sustainable development through afforestation. The model leverage plots in Fig. 2 illustrate the relationship between AFF, PG, GRQE, AP, and FDI with the Forest Area (FC).

Each variable's leverage value is displayed on the plot along with its impact on the Forest Area. The plot aids in identifying influential observations or outliers that may significantly impact the Forest Area variable. It offers information on how changes in each variable affect the Forest Area variable and potentially impact other variables as well. For instance, examining the leverage values for AFF, PG, and GRQE reveals that variations in these variables noticeably affect the Forest Area. This suggests that changes in agriculture, forestry, and fishing activities, population growth, and regulatory quality can influence the extent of forested areas. Similarly, the leverage values for AP and FDI indicate their impact on the Forest Area. Changes in average precipitation and FDI can also affect forested areas. By scrutinizing the leverage plot, we can identify influential observations or outliers that may significantly impact the Forest Area variable and potentially influence the relationships between the variables. This information is valuable for understanding the dynamics and interdependencies among these variables. Table 4 shows the RLS estimates.

**Table 4:** Robust least squares (RLS) regression.

Dependent Variable: FC				
Variables	Coefficient	Std. Error	z-Statistic	Prob.
AFF	0.190	0.078	2.426	0.015
PG	0.673	0.092	7.299	0.000
GRQE	1.901	0.759	2.502	0.012
AP	0.017	0.001	13.194	0.000
FDI	−0.020	0.154	−0.132	0.894
C	4.189	1.163	3.599	0.0003
**Robust Statistics**				
R-squared	0.666	Adjusted R-squared		0.605
Rw-squared	0.814	Adjusted Rw-squared		0.814

The results show the positive coefficient of AFF (i.e., 0.190) suggests a statistically significant positive relationship between the forest cover in Pakistan. This implies that economic activities in these sectors may be associated with afforestation efforts. Population growth emerges as a dominant factor, with a substantial positive coefficient of 0.673, indicating a significant increase in forest area with population growth. However, unexpected population growth has negative effects on natural resources, particularly forests. The study emphasizes the importance of community forest programs and controlled grazing in promoting an increase in forest cover. Regulatory quality, measured by GRQE, exhibits a positive impact on forest cover change, with a coefficient of 1.901, highlighting the crucial role of effective regulations. Additionally, average precipitation demonstrates a highly significant positive effect, emphasizing the role of climate conditions in influencing forest cover change. The study underscores the need to manage water resources and precipitation effectively for sustainable afforestation. The study calls for caution in determining how FDI may affect forest cover in Pakistan. According to pertinent studies, ecological restoration initiatives account for 56% of carbon sequestration in ecological restoration zones [69]. Therefore, maintaining sufficient forest cover is crucial for carbon sequestration. However, gender inequality persists in many government sectors and emerging nations. Encouraging women's involvement in the forest sector can be a quick strategy to increase forest cover and promote natural resources in Pakistan. Approximately 880 million people, mostly women, rely on forests for their home energy needs or as a source of income. Over the past three decades, global forest area has decreased by 4.2% (178 million ha) from 1990 to 2020 [70]. The World Bank estimates a potential loss of 18–20% of annual GDP by 2050 due to climate and environmental degradation [71]. Currently, 97% of Pakistan's fresh water is used for agriculture, representing 18% of the country's GDP [72]. The Punjab region, vital to Pakistan's economy, is susceptible to climate change impacts, including droughts, floods, and temperature increases [73].

Policies serve as guiding principles designed to facilitate the effective execution and attainment of goals. The government of Pakistan has implemented numerous policies in partnership with the forest department at different levels since 1955. There are ongoing endeavors to formulate policies and programs to increase the extent of wooded areas to fulfill the country's domestic requirements. Nevertheless, these efforts have yet to yield significant expansion in wooded regions; instead, there has been a discernible escalation in deforestation rates. One of the primary determinants is the substantial proportion of Pakistan's territory, estimated to be between 70 and 80%, that is situated in dry or semi-arid areas characterized by inadequate precipitation levels to sustain the development of trees [21]. Since forestry is within provincial authorities' jurisdiction, department experts mostly formulate policies about the forest sector. This approach is used to attain the required goals effectively. To advance the principles of sustainable development and secure advantages across economic, social, environmental, and cultural domains for current and future generations, the overarching objectives include the sustainable governance of forest resources [74]. The success of policy formation is only possible with the active participation of local people since including indigenous knowledge has significant value in the decision-making process. The successful execution of policies continues to provide a formidable obstacle, mainly attributable to political volatility, resource constraints, apathy, and power dynamics. These policies aim to enhance the preservation of natural resources by facilitating the growth of forested areas via reforestation and afforestation initiatives. Additionally, they seek to guarantee the fundamental requirements of individuals who rely on natural resources to achieve sustainable development. The goals established for restoration significantly influence government and institutional policies and operations [75]. The first forest policy was officially declared in 1955. It was subsequently followed by a series of further policies in 1962, 1975, 1980 (as an integral component of the national agricultural policy), 1988, 1991, 2001, the national Forest policy of 2002, the Forest Ordinance of 2022, and the most recent national forest policy in 2015. Despite implementing several programs, attaining significant forest cover has proven challenging, principally because of the scarcity of land suitable for afforestation. According to Ismail et al. [76], the Ministry of Climate Change has recently formulated a strategy plan to execute Pakistan's National Forest Monitoring System. The ongoing trend towards urbanization is a cause for concern. The obstacles encountered by the forest department should not be ascribed to their constraints in terms of resources and jurisdiction. The most recent strategy emphasizes the engagement of previously uninvolved stakeholders in planting initiatives. A contentious issue about eucalyptus planting operations has engendered disagreement between the forest department and non-governmental organizations (NGOs). There exists a divergence of opinions on the potential decrease in the water table. The recognition of biodiversity as a critical element occurred within the Pakistani government's approval of the National Conservation Strategy in 1992 [77]. When discussing the topic of sustainable development via afforestation, it is essential to recognize that this obligation extends beyond the purview of a single department and should be embraced by all individuals who reside on the planet. The alteration of forest cover is attributed to socioeconomic forces, which influence the composition and biodiversity of forest ecosystems. The New York Declaration on Forests garnered an endorsement from more than 100 governments, private enterprises, NGOs in 2014. This endorsement was in line with the objectives of the 2020 Bonn Challenge and aimed to expand reforestation efforts to cover an area of 350 million hectares by the year 2030 [78].

In this challenging scenario, where deforestation appears to be beyond the control of authorities, the efforts of the Forest Department are to be commended. It is plausible that certain elements, such as the timber industry, merely feign concern for forest preservation in a country like Pakistan, where effective deforestation control remains elusive. The recourse to recuperating lost ground lies in sustained afforestation and reforestation endeavors, although this undertaking is time-intensive. Sabir et al. [45] underscore the significance of afforestation as an environmental enhancement strategy, particularly in regions grappling with deforestation. The findings, consistent across two multi-criteria decision-making methods, underscore the applicability of this research for both local and international forestry departments, as well as researchers with an interest in the application of multi-criteria decision-making in forestry. Kamble et al. [79] findings elucidate the program's far-reaching impact, leading to substantial increases in precipitation and agricultural production encompassing both area and yield. These outcomes underscore the multifaceted benefits of afforestation, extending beyond carbon sequestration, and hint at its effectiveness as a climate mitigation strategy. Kousar et al. [80] conducted a study driven by the ongoing fluctuations in energy prices in Pakistan. The study uncovers significant positive associations between twin deficits and exchange rates with energy inflation, particularly with a more pronounced impact on oil prices. Additionally, urbanization, climate change, and energy production from oil and gas were found to positively influence electricity prices in the long run. These findings underscore the need for proactive policy interventions aimed at addressing energy inflation and its multifaceted societal implications. The research conducted by Ullah et al. [81] underscores the persistent challenge posed by population growth in the context of finite resources, urging the development of effective policies that align with the principles of sustainable development.

The statistical assessments confirm the credibility of our model, with R-squared, adjusted R-squared, and robust R-squared values indicating a good fit. The study's findings can inform policymakers and stakeholders in promoting sustainable development through afforestation, emphasizing the importance of sustainable agriculture, population management, regulatory reforms, and water resource management.

## 5 Conclusions and policy recommendations

We are grappling with the cries of a changing climate, underscoring the urgent need to protect natural resources for our survival and the well-being of future generations in Pakistan. The country maintains an average forest cover of 5.372%, ranging from 0.187% to 6.407%. This study, aimed at addressing key environmental and sustainable development concerns, has yielded valuable insights. Through correlation and robust least squares regression analysis, we've explored the multifaceted relationship between financial assistance, environmental regulations, precipitation, population growth, FDI inflows, and afforestation in Pakistan, shedding light on critical factors influencing the nation's environmental landscape. The research employed Robust Least Squares (RLS) regression to examine how forest cover in Pakistan from 1990 to 2022 was influenced by factors such as population dynamics, afforestation efforts, regulatory quality estimates, annual precipitation, and FDI. This study reiterates the primary research question: “Can sustainable development through afforestation in Pakistan be achieved, and what are the key factors influencing changes in forest cover?” To answer this question, we conducted comprehensive research that delved into the complex interplay of variables impacting Pakistan's forested areas. Our analysis, encompassing correlation and robust least squares regression results, has provided valuable insights into the multifaceted challenge of enhancing environmental quality and fostering afforestation in Pakistan. Population growth emerges as a dominant factor in our study, with a substantial positive coefficient affirming that as the population increases, there is a significant associated increase in forest area. However, this factor can also pose challenges, as the findings indicate a declining trend in forest cover over the decades. Effective regulatory quality is revealed as a pivotal factor positively impacting forest cover change. The coefficient of 1.901 emphasizes the importance of robust regulatory frameworks and governance practices in fostering afforestation initiatives, underscoring the significance of governance in Pakistan's sustainable development efforts. Average precipitation is another significant variable, exhibiting a highly positive effect, highlighting the importance of climate conditions in influencing forest cover. This finding underscores the need for water resource management and policy measures to ensure a consistent water supply, particularly in regions with diverse climatic conditions. Ultimately, it is imperative to prioritize sustainable agriculture and forest management, address population growth for sustainable development alongside forest conservation, emphasize regulatory reforms, and manage water resources effectively to promote sustainable afforestation. These data-driven recommendations are of significant importance for Pakistani policymakers and conservation initiatives working towards sustainable development in the country.

This study achieved its research objectives by assessing the synergistic impact of financial assistance and environmental regulations on afforestation, addressing adaptation strategies for variable precipitation levels and climate change, and investigating the influence of population growth rate and FDI on Pakistan's afforestation efforts. The findings provide a holistic understanding of the complex factors influencing sustainable development through afforestation in Pakistan, offering valuable insights for future conservation and policy initiatives.

- Short-Term Policy Recommendations
  1. Implement community-driven afforestation projects in collaboration with local stakeholders to ensure the active involvement of communities in planting and protecting trees, thereby accelerating reforestation efforts.
  2. Introduce short-term employment programs in forestry and conservation for rural communities, offering them opportunities to participate in afforestation activities while developing relevant skills.
  3. Launch nationwide tree-planting campaigns to raise public awareness about the importance of afforestation and engage citizens in tree-planting activities, promoting a culture of environmental responsibility.
  4. Strengthen forest protection and anti-illegal logging enforcement mechanisms, including increased patrols and monitoring of protected areas to curb deforestation and unauthorized woodcutting.
  5. Enhance data collection, research, and monitoring of forest cover changes to continually assess the effectiveness of ongoing afforestation programs and adjust policies accordingly.
  6. Develop district-specific Climate Change Adaptation Action Plans for every district across Pakistan.
- Medium-Term Policy Recommendations
  1. Develop comprehensive land-use and zoning policies that differentiate forested areas and promote sustainable land management practices to safeguard existing forests and encourage afforestation.
  2. Promote eco-tourism initiatives in forested regions, generating income for local communities while emphasizing the conservation of biodiversity, indirectly contributing to forest preservation.
  3. Implement gender-inclusive policies that empower women in forestry and conservation, recognizing their vital role in forest management and the sustainable use of forest resources.
  4. Develop afforestation strategies that consider climate change adaptation, including the planting of climate-resilient tree species, to ensure the longevity of new forested areas.
  5. Encourage the adoption of sustainable agricultural practices that minimize deforestation for agricultural expansion, promoting agroforestry and other eco-friendly farming techniques.
- Long-Term Policy Recommendations
  1. Develop long-term educational programs to ensure maximum climate literacy, environmental consciousness, and conservation values in future generations, fostering a sustained commitment to forest protection.
  2. Ensure the implementation of forest policies that enable rural communities to diversify their livelihoods beyond dependence on forest resources, reducing pressure on forests while improving their economic well-being.
  3. Establish large-scale, long-term afforestation projects targeting degraded landscapes and unused lands, with a focus on restoring and expanding forested areas.
  4. Ensure transparency and good governance in forest landscape restoration by strengthening legal frameworks, reducing corruption, and promoting community participation in decision-making.
  5. Foster international partnerships and collaborations to access funding, technology, and knowledge exchange, facilitating long-term afforestation projects and sustaining momentum for forest conservation.

These findings provide valuable insights for policymakers and stakeholders dedicated to promoting sustainable development through afforestation, fostering both environmental conservation and socioeconomic progress in Pakistan. The tailored policy recommendations, designed for different timeframes, aim to address the intricate challenge of forest cover change while advancing sustainable development through afforestation. Considering the multifaceted nature of the issue, these recommendations offer a holistic approach to safeguarding Pakistan's forested areas for future generations. The profound implications of these recommendations resonate with Pakistani policymakers and conservationists actively working towards sustainable development. In conclusion, this study unravels the complex web of factors shaping Pakistan's environmental landscape, offering vital insights for a greener and more sustainable future.

## Data availability statement

Data will be made available on request.

## CRediT authorship contribution statement

**Muhammad Asif Khan:** Writing – review & editing, Writing – original draft, Validation, Software, Methodology, Investigation, Formal analysis, Data curation, Conceptualization. **Sajid Ali:** Writing – review & editing, Writing – original draft, Visualization, Validation, Resources, Project administration, Investigation, Funding acquisition, Formal analysis, Data curation, Conceptualization. **Muhammad Khalid Anser:** Writing – review & editing, Writing – original draft, Visualization, Validation, Resources, Project administration, Investigation, Funding acquisition, Formal analysis, Data curation, Conceptualization. **Abdelmohsen A. Nassani:** Writing – review & editing, Writing – original draft, Validation, Supervision, Software, Resources, Project administration, Investigation, Funding acquisition, Formal analysis, Data curation, Conceptualization. **Khalid M. Al-Aiban:** Writing – review & editing, Writing – original draft, Visualization, Validation, Resources, Project administration, Investigation, Formal analysis, Data curation, Conceptualization. **Shafiq ur Rahman:** Writing – review & editing, Writing – original draft, Visualization, Validation, Resources, Project administration, Investigation, Formal analysis, Data curation, Conceptualization. **Khalid Zaman:** Writing – review & editing, Writing – original draft, Visualization, Validation, Supervision, Software, Resources, Project administration, Methodology, Investigation, Funding acquisition, Formal analysis, Data curation, Conceptualization.

## Declaration of competing interest

The authors declare that they have no known competing financial interests or personal relationships that could have appeared to influence the work reported in this paper.
